# Endovascular Management of Vertebrobasilar Trunk Artery Large Aneurysms: Complications and Long-Term Results

**DOI:** 10.3389/fneur.2022.839219

**Published:** 2022-02-18

**Authors:** Qiaowei Wu, Shancai Xu, Chunlei Wang, Zhiyong Ji, Yuchen Li, Bowen Sun, Yuxiao Meng, Huaizhang Shi, Pei Wu

**Affiliations:** Department of Neurosurgery, The First Affiliated Hospital of Harbin Medical University, Harbin, China

**Keywords:** vertebrobasilar artery, large aneurysms, endovascular treatment, complications, follow-up

## Abstract

**Objective:**

To analyze the complications and long-term results of endovascular management of vertebrobasilar trunk large (≥10 mm) aneurysms (VBTLAs) and identify predictors of outcomes.

**Methods:**

Between 2014 and 2020, 6,987 patients with intracranial aneurysms were referred to our center for aneurysm management and 2,224 patients have undergone the endovascular procedures. We retrospectively reviewed the database and identify all the patients with VBTLAs.

**Results:**

A total of 62 VBTLAs were identified. The median aneurysm size was 13.4 mm [interquartile range (IQR) 11.5–18.7]. Among them, 24 aneurysms were treated with overlapping stent techniques, 18 aneurysms were treated with flow diversion, 14 aneurysms were treated with single stent-assisted coiling, and 6 aneurysms were treated with coiling alone. Ten patients were treated with parent artery occlusion or unilateral vertebral artery occlusion. Periprocedural complications were occurred in 7 (11.3%) patients. Clinical follow-up was obtained at the median of 27.5 months (IQR 15.3–58.5). The overall complication rate was 16.1% (10/62), including nine ischemic events and one hemorrhagic event. The combined disability and neurological mortality rate was 12.9% (8/62), with 4 (6.5%) deaths. The favorable outcome rate at follow-up was 87.1% (54/62). The complication-free cumulative survival rates at 1 and 5 years were 86.8 and 82.0%, respectively. The overall cumulative survival rates at 1 and 5 year were 96.5 and 89.8%, respectively. In the multivariate Cox regression analysis, longer procedure time (>115 min) (*P* = 0.037) and ischemic onset (*P* = 0.005) predict complications. Angiography follow-up was available for 36 patients at the median of 6.0 months (IQR 6–12), with a complete occlusion rate of 77.8% (28/36). Two (5.6%) aneurysms were recanalized and subsequently received the retreatment. Subgroup analysis did not find any differences in the complete occlusion rate between endovascular strategies.

**Conclusion:**

Endovascular management of VBTLAs has a reasonable safety profile with favorable 5-year cumulative survival rates and imaging outcomes at follow-up. Prolonged procedure and ischemic onset are associated with a high risk of overall complications.

## Introduction

Posterior circulation aneurysms are always associated with a poor natural history and published reports have shown such aneurysms have a greater tendency to rupture than those in anterior circulation (1–3). Once ruptured, posterior circulation aneurysms are associated with a high mortality rate and poor prognosis within the first 48 h (4). Among the overall posterior circulation aneurysms, aneurysms located in the vertebrobasilar trunk artery are rare and limited data have existed on the epidemiology (3, 5), especially vertebrobasilar trunk large (≥10 mm) aneurysms (VBTLAs). If untreated, ischemic stroke and hemorrhage could lead to fatal results. Surgery is the essential management in those cases, including open surgery or endovascular procedures (3, 5). Endovascular management including coiling, stent-assisted coiling, and flow diversion (FD) has certain advantages compared to open surgery, but VBTLA is still associated with an increased complication risk as compared to aneurysms located in anterior circulation, due to their location, surgical accessibility, pathological features, and relation to perforating branches of vertebrobasilar artery (6–8). However, there are limited reports on the endovascular management of VBTLAs and the long-term clinical prognosis still needs to be further confirmed. In this study, we reviewed the database and described the single-center results of the epidemiology, clinical prognosis, and imaging outcomes of endovascular management of VBTLAs.

## Subjects and Methods

### Subjects

**Figure 1:**
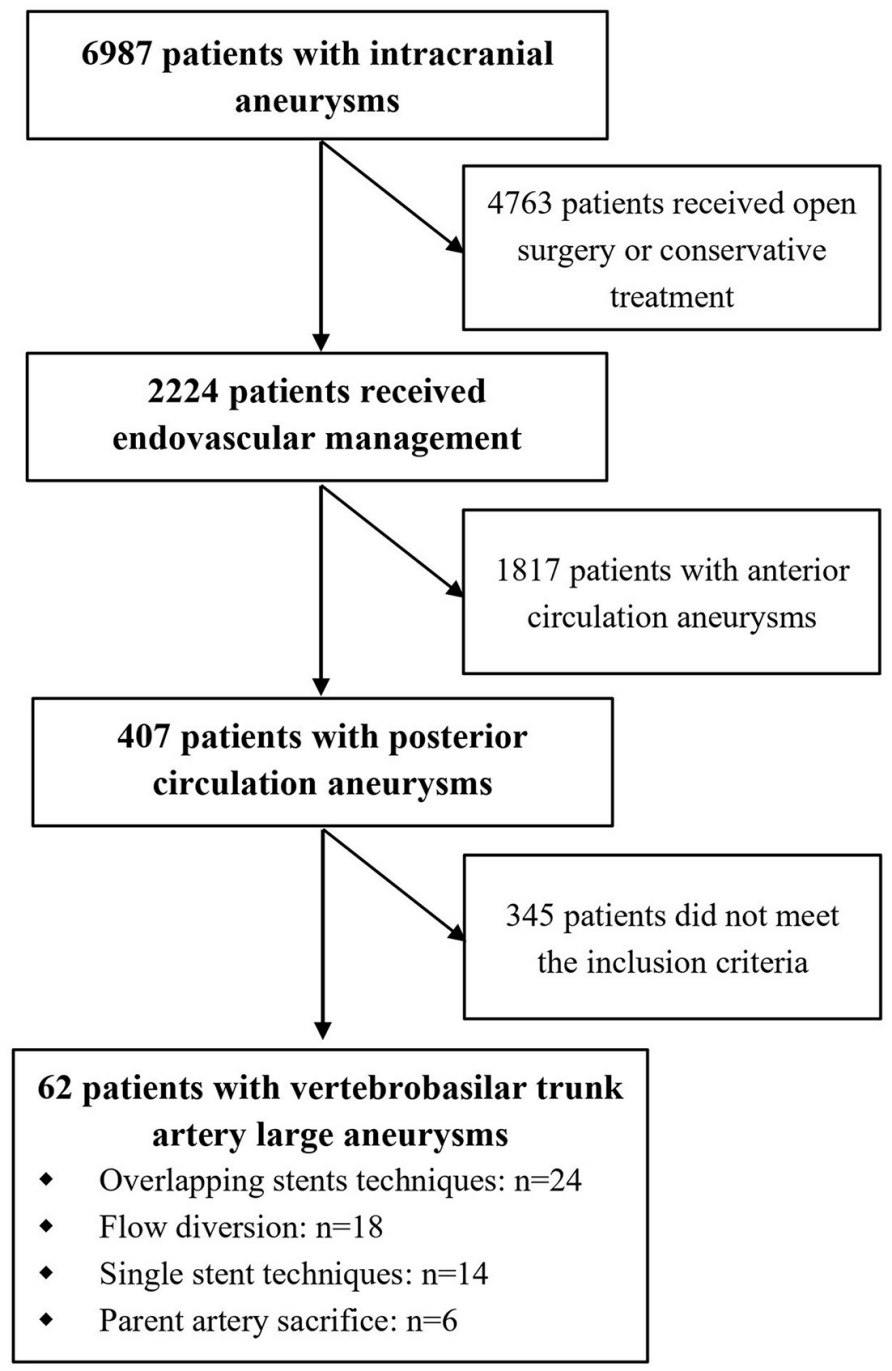
Flowchart of the patient selection process.

Between January 2014 and December 2020, 6,987 patients harboring intracranial aneurysms were referred to our center for aneurysm management and 2,224 patients have undergone the endovascular procedures (Figure 1). The institutional review board of the First Affiliated Hospital of Harbin Medical University approved this retrospective study and written informed consent was obtained from all the patients before the procedure.

Vertebrobasilar trunk large aneurysm is defined as a large (measuring ≥ 10 mm in diameter) aneurysm originating from an arterial segment of vertebral artery up to the origin of the superior cerebellar artery (5). By definition, aneurysms originating from the perforating branch of the vertebrobasilar artery and basilar tip aneurysms were excluded. In addition, the modified Rankin Scale (mRS) > 2 before the symptom onset and patients complicated by other treated intracranial aneurysms were also excluded to avoid potential influences on the results.

### Procedures

The decision to treat VBTLAs with endovascular management was based on the aneurysm morphology and the relation to perforating branches of the vertebrobasilar artery. The doctors informed the patients of all the treatment options, after which the patients individually discussed with their doctors and chose the final treatment protocol. In patients with insufficient collateral circulation and favorable geometry, coiling of the aneurysm with maintaining of the parent artery patency was performed. Wide-neck aneurysms were coiled with stent-assisted by the low-profile visualized intraluminal support (LVIS) device (MicroVention-Terumo, Aliso Viejo, CA, USA), Enterprise (Codman Neurovascular, Raynham, MA, USA), and Solitaire (ev3, Irvine, CA, USA). According to the anatomic factors of the aneurysms and the adjacent vessels, parent artery occlusion or unilateral vertebral artery occlusion was performed at the discretion of the neurointerventionists. The balloon occlusion test (BOT) was performed to test the patient tolerance for permanent artery occlusion before the procedure. For patients who remained neurologically intact during the BOT, artery occlusion was then performed with coils alone or with a combination of Onyx. For patients who chose to be treated with FD, the pipeline embolization device (PED) (Covidien, Irvine, CA, USA) was deployed with or without adjunctive coiling.

### Antiplatelet Therapy

For all the patients who were treated by stent-assisted coiling or FD, dual antiplatelet therapy (aspirin 100 mg/clopidogrel 75 mg) was administrated at least 3–5 days before the procedure and clopidogrel response was studied by using thromboelastography (TEG). Intraprocedural tirofiban usage or a preprocedural loading dose of 300 mg clopidogrel and 300 mg aspirin was administrated in patients with ruptured aneurysms. Dual antiplatelet therapy was maintained for at least 3 months (6 months for patients treated with FD) after the stent deployment, followed by 100 mg aspirin daily, indefinitely.

### Clinical Features

The initial onset was classified as ischemic symptoms, hemorrhage, or other presentations including headache, mass effect, and incidental. Baseline characteristics of patients, including sex, age, hypotension, diabetes mellitus, smoking, and alcohol abuse. Baseline characteristics of aneurysms, including location, size, morphology, and incorporation of a branch vessel. Endovascular procedure-related data, complications, and patient neurological status were retrospectively collected and analyzed. Clinical follow-up was assessed by the mRS score, while the Raymond–Roy grading scale was used to evaluate the aneurysm occlusion status (9). The follow-up mRS score ≤ 2 was defined as a favorable outcome and grade 1 of the Raymond–Roy grading scale was defined as complete occlusion of the aneurysm.

### Statistical Analysis

In descriptive statistics, qualitative variables were presented as numbers followed by percentages. Continuous and normally distributed variables were presented as mean ± SD and continuous and non-normally distributed variables were presented as the median and interquartile range (IQR). The Kaplan–Meier survival analysis was used to calculate the complication-free cumulative survival rates and the overall cumulative survival rates at follow-up. The univariate and multivariate Cox regression analysis was performed to identify the independent risk factors for overall treatment-related complications. *P* < 0.05 was considered to indicate statistical significance. Statistical analysis was performed by using SPSS software version 22.0 (IBM SPSS Incorporation, Chicago, IL, USA).

## Results

### Baseline Characteristics and Procedure-Related Data

Among the 2,224 patients, 407 (18.3%) patients harboring 432 posterior circulation aneurysms were identified (Figure 1). A total of 62 (2.8%) patients in our population harboring 62 VBTLAs (12 ruptured and 50 unruptured) were identified and enrolled in this study. The cohort comprised 22 (35.5%) women and 40 (64.5%) men, with a mean age of 55.8 ± 8.7 (range: 39–79) years. The median aneurysm size was 13.4 mm (IQR 11.5–18.7). A total of 44 (71.0%) aneurysms were originated from the V4 segment of the vertebral artery, 13 (21.0%) aneurysms were originated from the basilar artery, and 5 (8.0%) aneurysms were originated from the vertebrobasilar junction. Thirteen (21.0%) aneurysms involved side branches, including 2 (3.2%) aneurysms involving the anterior inferior cerebellar artery (AICA) and 11 (17.7%) aneurysms involving the posterior inferior cerebellar artery (PICA). The detailed baseline characteristics of patients and aneurysms are shown in Table 1.

**Table 1:** Baseline characteristics of patients and aneurysms.

**Characteristics**	***n* = 62 patients (62 aneurysms)**
Male, *n* (%)	40 (64.5)
Mean age, years (±SD)	55.8 ± 8.7
Risk factors, *n* (%)	
Hypertension	34 (54.8)
Diabetes mellitus	4 (6.5)
Smoking	19 (30.6)
Alcohol abuse	20 (32.3)
Median maximum diameter of aneurysm, mm (IQR)	13.4 (11.5-18.7)
Onset Symptoms, *n* (%)	
Ischemic stroke	11 (17.7)
Hemorrhage	12 (19.4)
Others	39 (62.9)
Aneurysm location, *n* (%)	
BA	13 (21.0)
VBJ	5 (8.0)
VA	44 (71.0)
Aneurysm shape, *n* (%)	
Saccular	3 (4.8)
Fusiform and/or dissecting	59 (95.2)
Ruptured aneurysm, *n* (%)	12 (19.4)
Aneurysms involving side branches, *n* (%)	13 (21.0)

*IQR, interquartile range; BA, basilar artery; VBJ, vertebrobasilar junction; VA, vertebral artery*.

Of the 62 aneurysms, 24 (38.7%) aneurysms were treated with overlapping stents techniques, 18 (29.0%) aneurysms were treated with FD, 14 (22.6%) aneurysms were treated with single stent-assisted coiling, and 6 (9.7%) aneurysms were treated with coiling alone. Artery sacrificed techniques were performed in 10 aneurysms, of which 6 (9.7%) aneurysms were treated with parent artery occlusion and 4 (6.5%) aneurysms were treated with unilateral vertebral artery occlusion. The median procedure time was 105.0 min (IQR 75.0–120.0). All the 12 ruptured aneurysms were treated within 24 h after admission. Endovascular procedure-related data are shown in Table 2.

**Table 2:** Endovascular procedure-related data.

**Procedures details**	***n* = 62 patients (62 aneurysms)**
**Endovascular treatment strategies**, ***n*** **(%)**	
Overlapping stents techniques	24 (38.7)
Series stent-assisted coiling	22 (35.5)
Series stent-assisted coiling and unilateral vertebral artery sacrifice	2 (3.2)
Single stents techniques	14 (22.6)
Single stent-assisted coiling	13 (21.0)
Single stent-assisted coiling and unilateral vertebral artery sacrifice	1 (1.6)
Flow diverter treatment	18 (29.0)
Flow diverter alone	17 (27.4)
Flow diverter implantation and unilateral vertebral artery sacrifice	1 (1.6)
Aneurysm coiling and parent artery sacrifice	6 (9.7)
Type of stent placed, *n* (%)	*n* = 85 stents
LVIS	31 (36.5)
Enterprise	33 (38.8)
Solitaire	1 (1.2)
Pipeline embolization device	20 (23.5)
Median procedure time, minutes (IQR)	105.0 (75.0–120.0)
Mean number of coils used, *n* = 45 (±SD)	9.6 ± 4.6

*IQR, interquartile range; LVIS, low-profile visualized intraluminal support*.

### Complications and Clinical Outcomes

Clinical follow-up was obtained at the median of 27.5 months (IQR 15.3–58.5). A total of 10 (16.1%) patients had complications, including 9 (14.5%) patients who had ischemic events and 1 (1.6%) patient with hemorrhagic event, of which 7 (11.3%) patients occurred during the periprocedural phase and 3 (4.8%) patients occurred during the follow-up (Table 3). The combined disability and neurological mortality rate was 12.9% (8/62), including 4 (6.5%) deaths. The favorable outcome rate at follow-up was 87.1% (54/62).

**Table 3:** Baseline characteristics of patients with postprocedural complications.

**Patient No**.	**Location**	**Type of stents**	**Complication description**		**mRS at discharge**	**mRS at last FU**
			**Peri**	**FU**		
1	VA	EP	–	One-sided motor weakness	0	3
2	VBJ	LVIS	Cerebellar hemorrhage	–	2	0
3	BA	EP + LVIS × 2	Persistent dizziness	–	1	1
4	VA	EP + LVIS	–	One-sided motor weakness	1	4
5	VBJ	EP × 2 + LVIS	Dysphagia	Acute cerebral infarctions	1	6
6	BA	EP + LVIS	One-sided motor weakness and dysphagia	–	4	4
7	VA	LVIS	Dysphagia	Acute cerebral infarctions	1	6
8	VBJ	PED	In-stent thrombosis	Acute cerebral infarctions	0	6
9	BA	PED	–	In-stent thrombosis	0	6
10	BA	EP+LVIS	One-sided motor weakness	–	4	4

*VA, vertebral artery; VBJ, vertebrobasilar junction; BA, basilar artery; EP, enterprise stent; LVIS, low-profile visualized intraluminal support; PED, pipeline embolization device; Peri, periprocedural complication; FU, follow-up; mRS, modified Rankin Scale*.

Among the seven periprocedural complications, one patient was treated for a vertebral aneurysm by using FD developed acute parent artery occlusion during the immediate post-procedural digital subtraction angiography (DSA) and catheter-directed thrombolysis with urokinase was then performed. The patient presented no neurological deficit after discharge, but the patient died due to the acute cerebral infarction during the follow-up. Two patients experienced dysphagia after the single-stent assisted coiling and overlapping stent-assisted coiling of the vertebral aneurysm and vertebrobasilar junction aneurysm, respectively. The mRS score at discharge was both 1. However, the two patients died during the follow-up, one of them died due to the pulmonary embolism after acute cerebral infarction, and the other patient died due to the complications after acute cerebral infarction. One patient treated for a basilar aneurysm with overlapping stent techniques presented dysphagia and two-sided motor weakness and following brain MRI demonstrated the acute cerebellar infarctions: the mRS score at discharge was 4. One patient presented persistent dizziness 5 days after the overlapping stent-assisted coiling of a basilar aneurysm and acute cerebellar infarctions were also noted in brain MRI: the mRS score at discharge was 1. One patient presented one-sided motor weakness after the overlapping stent-assisted coiling of a basilar aneurysm and the mRS score at discharge was 4. The other one patient treated for a vertebrobasilar junction aneurysm with single stent-assisted coiling developed a headache 3 days after the procedure and a CT scan showed cerebellar hemorrhage: the mRS score at discharge was 1.

**Figure 2:**
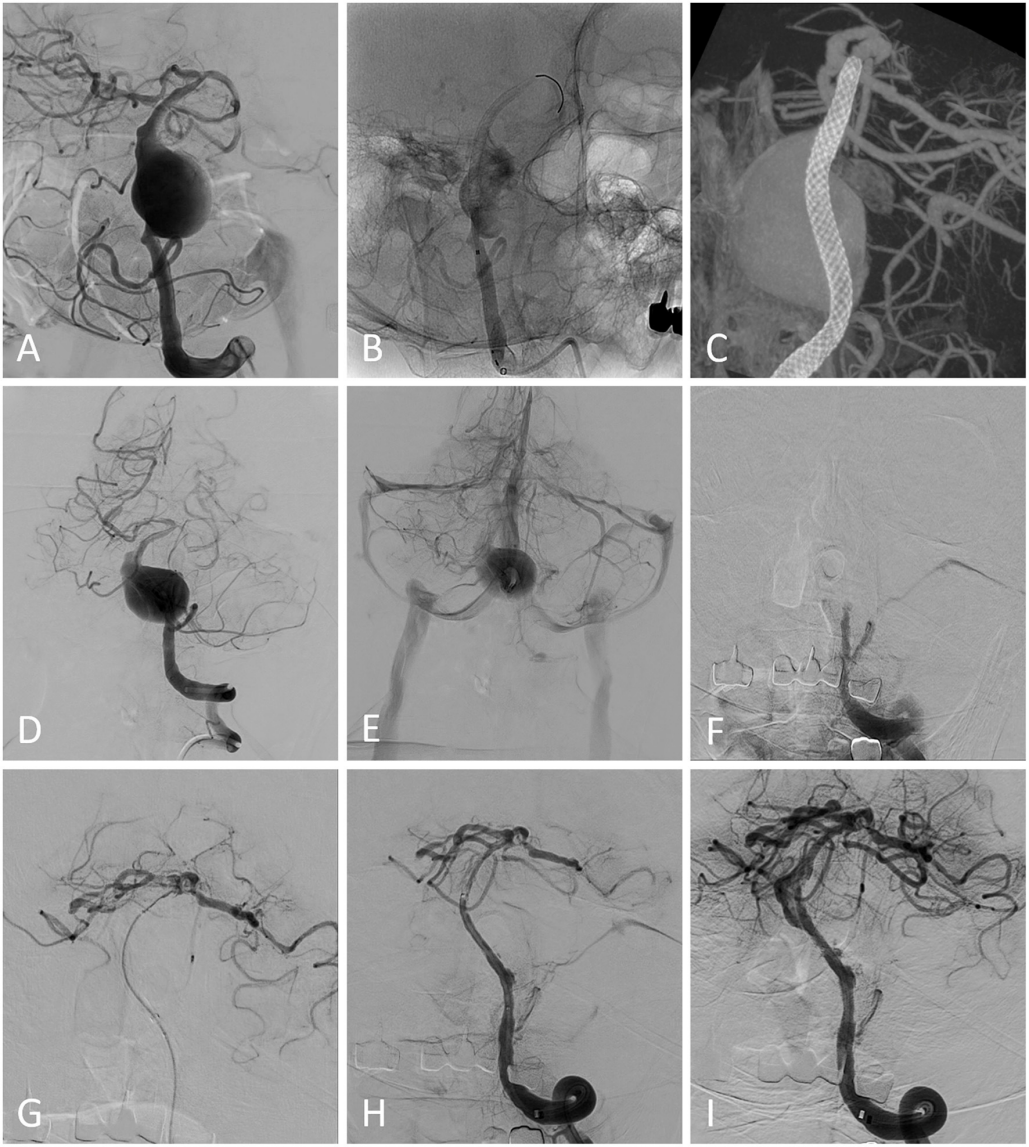
**(A)** Pretreatment image from a patient demonstrating a basilar artery aneurysm. **(B)** A pipeline embolization device was deployed without adjunctive coiling. The immediate postprocedural Vaso CT showed that the stent was deployed properly **(C)**. The postprocedural digital subtraction angiography (DSA) showed the patency of the parent artery **(D)** and the blood flow detained to the venous phase was detected **(E)**. The patient presented sudden nausea, vomiting, and progressive consciousness disorder 5 months after the procedure. Emergency DSA showed that the parent artery was occluded **(F)** and aspiration thrombectomy was performed **(G,H)**. Blood flow was restored after the procedure and the aneurysm neck residual was detected **(I)**. The patient died 1 day later.

A total of 3 (4.8%) patients experienced new-onset neurological deficits during the follow-up. One patient treated for a basilar artery aneurysm experienced in-stent thrombosis 5 months after the FD implantation. Despite the aspiration thrombectomy was performed, the patient died 1 day later (Figure 2). The other two patients treated for vertebral aneurysms presented one-sided motor weakness during the follow-up at 7 and 24 months, respectively. The mRS at the last follow-up was 3 and 4, respectively.

### Survival Analysis

The complication-free cumulative survival rates at 1- and 5-year were 86.8 and 82.0%, respectively. The overall cumulative survival rates at 1- and 5-year were 96.5 and 89.8%, respectively. The univariate Cox regression analysis showed that ischemic onset (*P* = 0.001), older age (≥60 years) (*P* = 0.054), aneurysms involving basilar artery (*P* = 0.006), and longer procedure time (>115 min) (*P* = 0.010) were related to overall complications. In the multivariate Cox regression analysis, longer procedure time (>115 min) [hazard ratio (HR): 7.12, 95% CI: 1.13–45.00; *P* = 0.037] and ischemic onset (HR: 10.06, 95% CI: 2.03–49.81; *P* = 0.005) were statistically significant risk factors associated with overall complications (Table 4).

**Table 4:** The univariate and multivariate Cox regression analysis for serious adverse events.

**Variable**	**Univariable**			**Multivariable**		
	**HR**	**95%CI**	***P*-value**	**HR**	**95%CI**	***P-*value**
Parent artery or unilateral vertebral artery sacrifice	2.22	0.60–9.02	0.223	–	–	–
Flow diversion	0.65	0.14–3.08	0.582	–	–	–
Aneurysms involving side branches	2.33	0.65–8.30	0.193	–	–	–
Procedure time (>115 min)	7.66	1.62–36.12	0.010	7.12	1.13–45.00	0.037
Age, (≥60 years)	3.47	0.98–12.32	0.054	2.82	0.76–10.51	0.122
Sex (male)	1.27	0.33–4.91	0.729	–	–	–
Aneurysms involved basilar artery	6.67	1.72–25.88	0.006	1.02	0.17–6.11	0.979
Hypertension	3.44	0.73–16.20	0.118	–	–	–
Ischemic onset	8.69	2.43–31.04	0.001	10.06	2.03–49.81	0.005
Diabetes mellitus	1.61	0.20–12.71	0.652	–	–	–
Smoking	0.54	0.12–2.56	0.440	–	–	–
Alcohol abuse	1.25	0.35–4.46	0.728	–	–	–

### Angiography Outcomes

Angiography follow-up was available for 36 aneurysms at the median of 6.0 months (IQR 6–12). Of the 36 aneurysms, 28 (77.8%) aneurysms demonstrating complete occlusion (grade 1 of the Raymond–Roy grading scale) and 34 (94.4%) aneurysms demonstrating adequate occlusion (grades 1 and 2 of the Raymond–Roy grading scale). Two (5.6%) aneurysms were recanalized and subsequently received the retreatment. Subgroup analysis did not find any differences (*P* = 0.886) in the complete occlusion rate between overlapping stent techniques (10/13), single stent techniques (6/7), FD (9/13), and parent artery sacrifice (3/3).

## Discussion

Specific epidemiological reports of VBTLAs are limited. The previous study has shown that the percentage of the aneurysm located in the vertebrobasilar or posterior cerebral artery system was 8.3% and demonstrated that such aneurysms were significantly associated with the rupture (10). Saliou et al. (5) showed that the percentage of basilar trunk artery aneurysms was 2.1% among the overall aneurysm locations. This study presented the percentage of VBTLAs among the overall aneurysm location was 2.8%, which indicated that VBTLAs are exceptionally rare. As shown by Saliou et al. (5) and Kai et al. (11), large or growing vertebrobasilar aneurysms are associated with an increased risk of bleeding and early surgical management should be considered. Once the rupture of vertebrobasilar dissecting aneurysms occurs, the rerupture rate is high (up to 70%) and more than half of the rerupture occurs within 24 h (1). Flemming et al. (12) studied the natural history of vertebrobasilar non-saccular aneurysms and found that the mortality and morbidity were 40 and 53% during the follow-up, respectively, with the median survival of 7.8 years. Due to the high risk of rupture and poor natural history of VBTLAs, once detected, it is important to individualize surgical intervention on the basis of the overall medical condition of the patient (1, 11–13). For patients with aneurysmal subarachnoid hemorrhage, aneurysm obliteration should be performed as soon as the patient is medically stabilized because of rebleeding (1, 13). In this study, the 5-year complication-free and overall cumulative survival rates were 82.0 and 89.8%, respectively, which indicated that the surgical intervention could benefit patients with vertebrobasilar aneurysms.

Open surgery had been attempted for treating large/giant posterior circulation aneurysms, including clipping, trapping, vessel occlusion, and bypass (14–16). But, because of the limited surgical accessibility of open surgery and the relation to perforating branches, patients with posterior circulation aneurysms are associated with higher morbidity and mortality as compared to those with anterior circulation aneurysms (15). Endovascular strategies, such as coiling, single stent-assisted coiling, overlapping stent-assisted coiling, parent artery sacrifice, and FD, which have been increasingly used for treating such aneurysms, can avoid the extensive surgical invasion and cranial nerve deficits. However, VBTLAs still pose a challenge to neurointerventionists with a relatively high complication rate. Liang et al. (17) reported 99 patients harboring 103 large/giant posterior circulation aneurysms, including 88 vertebrobasilar aneurysms, and showed an overall periprocedural complication rate of 17.2%. Chalouhi et al. (18) analyzed the complications of 334 coiled large/giant aneurysms and found a trend that posterior circulation aneurysms could predict complications and recurrence. In this study, we demonstrated an overall complication rate of 16.1% (10/62), which was similar to the complication rates of overall posterior circulation aneurysms in published studies (7, 17–20).

In a systematic review of posterior cerebral artery aneurysms treated by endovascular management, Sturiale et al. (20) found that the overall ischemic complication rate was 15%, while the other systematic review reported that the rate of ischemic event was 11.0% in FD treatment of posterior circulation aneurysms (19). In this study, we presented an ischemic event rate of 14.5% (9/62) in the endovascular management of VBTLAs, which indicates that the result was similar to the rate in the overall posterior circulation aneurysm cohort. The specific mechanisms of such events are not yet clarified. Two patients treated with FD experienced the in-stent thrombosis, of which one occurred intraprocedurally and the other occurred during the follow-up. Improper stent expansion could be the possible reason for the thrombosis right after stenting (21). In addition, irregular antiplatelet drug usage and delayed cobalt allergic reaction may also be the possible cause for the delayed in-stent thrombosis (21, 22). Two patients were treated with overlapping stent-assisted coiling with concomitant unilateral vertebral artery sacrificing. Even though the BOT was performed before the occlusion, patients with the negative BOT are still at risk for ischemia (23). The other five patients treated with stent-assisted coiling experienced ischemic events during the periprocedural phase or delayed ischemic events during the follow-up. The acute or delayed perforating branch occlusion, *in-situ* thrombus formation within the stent, and the detachment of vessel mural thrombus or intraluminal thrombus during the stenting or coiling may also be potential causes for ischemic events after the endovascular management.

Hemorrhagic events are relatively uncommon. Liang et al. (17) reported a hemorrhagic event rate of 2.0% in the cohort of 103 large/giant posterior circulation aneurysms and Alwakeal et al. (19) demonstrated a hemorrhagic complication of 3.4% in their systematic review. In this study, only 1 (1.6%) patient presented sudden headaches 3 days after the embolization and the cerebellar hemorrhage was found in the following CT scan. The exact mechanisms are not yet known, but multiple causes could contribute to hemorrhage. Intraprocedural microvascular embolization of various materials, including microthrombus, catheter coating, and air bubbles that result in vessel damage or ischemia-reperfusion hemorrhage (22). Moreover, published studies have demonstrated that antiplatelet hyperresponsiveness is associated with hemorrhagic events after the stenting (24). In addition, a history of hypertension may also be a potential cause.

In this study, we found that the ischemic onset was associated with a high risk of overall complications after the procedures (*P* = 0.005). Flemming et al. (12) found that the history of prior ischemia due to vertebrobasilar non-saccular aneurysms was a predictor of cerebral ischemia after the procedure of giant vertebrobasilar aneurysms. For patients with an initial ischemic onset, the risk of ischemic stroke recurrence was 6.7% per year and the median time to a second ischemic stroke was 1.73 years. The risk of ischemic stroke associated with the aneurysm increased from 2.7 at 1 year to 15.9% at 10 years. Intra-aneurysmal partially thrombosis is common in VBTLAs and the intra-aneurysmal thrombus detachment may cause occlusion of perforating vessels or distal vessels, leading to ischemic stroke. Also, the relatively low blood flow of perforating vessels after the procedure and the *in-situ* thrombosis within the parent artery or within perforating vessels may also lead to ischemia. In addition, this study showed that the prolonged procedure time might be associated with a high risk for overall complications after the procedures (*P* = 0.034). The long procedure time means a long vascular mechanical manipulation, which is associated with high risks of vascular endothelial injury, platelet activation, and arterial plaques detachment (22).

This study demonstrated a relatively low rate of complete occlusion (77.8%) for VBTLAs treated with endovascular management. Intra-aneurysmal thrombus resolution and coil migration into the thrombus mass may attribute to the aneurysm residual or the aneurysm recurrence (18). Previous studies have reported that the endovascular treatment of large or giant vertebrobasilar aneurysms was related to relatively unfavorable angiography outcomes, with higher recurrence and retreatment rates as compared to aneurysms located in anterior circulations (18, 25, 26). Our results were similar to those of Liang et al. (17), who reported a complete occlusion rate of 74.1% at the median follow-up duration of 6.8 months, in the cohort of 103 large or giant posterior circulation aneurysms treated with endovascular management. Despite a relatively low complete occlusion rate, we did not find the occurrence of aneurysm rupture or rerupture after the procedures, which indicated a beneficial in preventing aneurysmal hemorrhage.

There are some limitations. Due to the selection bias, the percentage of VBTLAs among the overall aneurysm location in this study could not represent the incidence in the real-world population and the incidence in the real world may be lower. In addition, 26 (41.9%) aneurysms were lost to angiography follow-up, which might have biased our findings. Moreover, this study was retrospective and the potential bias inherent to retrospective studies is unavoidable. Therefore, prospective studies with adequate long-term follow-ups are needed.

## Conclusion

Vertebrobasilar trunk large aneurysms are rare. For patients with VBTLAs, endovascular management seems to have a reasonable safety profile with favorable 1- and 5- year cumulative survival rates and angiography outcomes at the follow-up. In addition, it should be noted that prolonged procedure and ischemic onset are associated with a high risk of overall complications.

## Data Availability Statement

The raw data supporting the conclusions of this article will be made available by the authors, without undue reservation.

## Ethics Statement

The studies involving human participants were reviewed and approved by the Institutional Review Board of the First Affiliated Hospital of Harbin Medical University. The patients/participants provided their written informed consent to participate in this study. Written informed consent was obtained from the individual(s) for the publication of any potentially identifiable images or data included in this article.

## Author Contributions

QW, SX, HS, and PW contributed to study conception and design. QW, SX, CW, ZJ, YL, BS, and YM contributed to data acquisition, data interpretation, and analysis. QW and PW drafted the manuscript. HS contributed to the major revision of the manuscript. HS, PW, SX, CW, and ZJ contributed to the significant intellectual content. All authors made a significant contribution to this study, manuscript preparation, critically revised the manuscript, and approved the final version of the manuscript.

## Funding

This study was supported by grants from the National Natural Science Foundation (81901190) and Natural Science Foundation of Heilongjiang Province of China (Q2019H015).

## Conflict of Interest

The authors declare that the research was conducted in the absence of any commercial or financial relationships that could be construed as a potential conflict of interest.

## Publisher's Note

All claims expressed in this article are solely those of the authors and do not necessarily represent those of their affiliated organizations, or those of the publisher, the editors and the reviewers. Any product that may be evaluated in this article, or claim that may be made by its manufacturer, is not guaranteed or endorsed by the publisher.

## References

[1] Mizutani T Aruga T Kirino T Miki Y Saito I Tsuchida T. Recurrent subarachnoid hemorrhage from untreated ruptured vertebrobasilar dissecting aneurysms. Neurosurgery. (1995) 36:905–11; discussion 12–3. 10.1227/00006123-199505000-00003 7791980

[2] Sonobe M Yamazaki T Yonekura M Kikuchi H. Small unruptured intracranial aneurysm verification study: SUAVe study, Japan. Stroke. (2010) 41:1969–77. 10.1161/STROKEAHA.110.585059 20671254

[3] Wiebers DO. Unruptured intracranial aneurysms: natural history, clinical outcome, and risks of surgical and endovascular treatment. Lancet. (2003) 362:103–10. 10.1016/s0140-6736(03)13860-3 12867109

[4] Schievink WI Wijdicks EF Piepgras DG Chu CP O'Fallon WM Whisnant JP. The poor prognosis of ruptured intracranial aneurysms of the posterior circulation. J Neurosurg. (1995) 82:791–5. 10.3171/jns.1995.82.5.0791 7714604

[5] Saliou G Sacho RH Power S Kostynskyy A Willinsky RA Tymianski M. Natural history and management of basilar trunk artery aneurysms. Stroke. (2015) 46:948–53. 10.1161/STROKEAHA.114.006909 25712945

[6] Algra AM Lindgren A Vergouwen MDI Greving JP van der Schaaf IC van Doormaal TPC. Procedural clinical complications, case-fatality risks, and risk factors in endovascular and neurosurgical treatment of unruptured intracranial aneurysms: a systematic review and meta-analysis. JAMA Neurol. (2019) 76:282–93. 10.1001/jamaneurol.2018.4165 30592482 PMC6439725

[7] Domingo RA Tripathi S Perez-Vega C Vivas-Buitrago T Lu VM Todnem ND. Treatment of posterior circulation non-saccular aneurysms with flow diversion versus stent-assisted coiling: a systematic review and meta-analysis. J Neurointerv Surg. (2021) 13:159–63. 10.1136/neurintsurg-2020-016294 32651184

[8] Molyneux AJ Kerr RSC Yu L-M Clarke M Sneade M Yarnold JA. International subarachnoid aneurysm trial (ISAT) of neurosurgical clipping versus endovascular coiling in 2143 patients with ruptured intracranial aneurysms: a randomised comparison of effects on survival, dependency, seizures, rebleeding, subgroups, and aneurysm occlusion. Lancet. (2005) 366:809–17. 10.1016/s0140-6736(05)67214-5 16139655

[9] Roy D Milot G Raymond J. Endovascular treatment of unruptured aneurysms. Stroke. (2001) 32:1998–2004. 10.1161/hs0901.095600 11546888

[10] Investigators ISoUIA. Unruptured intracranial aneurysms–risk of rupture and risks of surgical intervention. N Engl J Med. (1998) 339:1725–33. 10.1056/nejm199812103392401 9867550

[11] Kai Y Nishi T Watanabe M Morioka M Hirano T Yano S. Strategy for treating unruptured vertebral artery dissecting aneurysms. Neurosurgery. (2011) 69:1085–91; discussion 91–2. 10.1227/NEU.0b013e3182262adf 21629133

[12] Flemming KD Wiebers DO Brown RD. Jr, Link MJ, Huston J, 3rd, McClelland RL, et al. The natural history of radiographically defined vertebrobasilar nonsaccular intracranial aneurysms. Cerebrovasc Dis. (2005) 20:270–9. 10.1159/000087710 16123548

[13] Tawk RG Hasan TF D'Souza CE Peel JB Freeman WD. Diagnosis and treatment of unruptured intracranial aneurysms and aneurysmal subarachnoid hemorrhage. Mayo Clin Proc. (2021) 96:1970–2000. 10.1016/j.mayocp.2021.01.005 33992453

[14] Nanda A Sonig A Banerjee AD Javalkar VK. Microsurgical management of giant intracranial aneurysms: a single surgeon experience from Louisiana State University, Shreveport. World Neurosurg. (2014) 81:752–64. 10.1016/j.wneu.2012.12.010 23246634

[15] Sughrue ME Saloner D Rayz VL Lawton MT. Giant intracranial aneurysms: evolution of management in a contemporary surgical series. Neurosurgery. (2011) 69:1261–70; discussion 70–1. 10.1227/NEU.0b013e31822bb8a6 21734614 PMC3529163

[16] Tjahjadi M Niemela M Kivelev J Serrone J Maekawa H Jahromi BR. Presigmoid approach to vertebrobasilar artery aneurysms: a series of 31 patients and review of the literature. World Neurosurg. (2016) 92:313–22. 10.1016/j.wneu.2016.05.001 27185653

[17] Liang F Zhang Y Yan P Ma C Liang S Jiang P. Predictors of periprocedural complications and angiographic outcomes of endovascular therapy for large and giant intracranial posterior circulation aneurysms. World Neurosurg. (2019) 125:e378–84. 10.1016/j.wneu.2019.01.080 30703589

[18] Chalouhi N Tjoumakaris S Gonzalez LF Dumont AS Starke RM Hasan D. Coiling of large and giant aneurysms: complications and long-term results of 334 cases. AJNR Am J Neuroradiol. (2014) 35:546–52. 10.3174/ajnr.A3696 23945229 PMC7964717

[19] Alwakeal A Shlobin NA Golnari P Metcalf-Doetsch W Nazari P Ansari SA. Flow diversion of posterior circulation aneurysms: systematic review of disaggregated individual patient data. AJNR Am J Neuroradiol. (2021) 42:1827–33. 10.3174/ajnr.A7220 34385140 PMC8562750

[20] Sturiale CL De Waure C Della Pepa GM Calabro GE Albanese A D'Argento F. Endovascular treatment of the posterior cerebral artery aneurysms: single-center experience and a systematic review. World Neurosurg. (2016) 91:154–62. 10.1016/j.wneu.2016.03.083 27062918

[21] Fujii S Fujita K Yamaoka H Miki K Hirai S Nemoto S. Refractory in-stent stenosis after flow diverter stenting associated with delayed cobalt allergic reaction. J Neurointerv Surg. (2021). 10.1136/neurintsurg-2021-017948 34433645 PMC8938677

[22] Wu Q Shao Q Li L Liang X Chang K Li T. Prophylactic administration of tirofiban for preventing thromboembolic events in flow diversion treatment of intracranial aneurysms. J Neurointerv Surg. (2021) 13:835–40. 10.1136/neurintsurg-2020-016878 33199539

[23] Ma Y Zhang Y Jiang J Li S Ni T Liu L. Application and reliability of the superselective balloon occlusion test in the treatment of complex cerebral artery aneurysms: a report of 12 cases. J Clin Neurosci. (2019) 64:57–63. 10.1016/j.jocn.2019.04.016 31029527

[24] Tonetti DA Jankowitz BT Gross BA. Antiplatelet therapy in flow diversion. Neurosurgery. (2020) 86:S47–52. 10.1093/neuros/nyz391 31838537

[25] Li M Liang H Wang J. Unfavorable outcomes related to endovascular treatment of giant vertebrobasilar aneurysms. Front Neurol. (2020) 11:748. 10.3389/fneur.2020.00748 32849210 PMC7431816

[26] Mu S Li C Yang X Wang Y Li Y Jiang C. Reconstructive endovascular treatment of spontaneous symptomatic large or giant vertebrobasilar dissecting aneurysms: clinical and angiographic outcomes. Clin Neuroradiol. (2016) 26:291–300. 10.1007/s00062-014-0369-4 25540817

